# A randomized controlled trial of tai chi for long-term low back pain (TAI CHI): Study rationale, design, and methods

**DOI:** 10.1186/1471-2474-10-55

**Published:** 2009-05-28

**Authors:** Amanda M Hall, Chris G Maher, Jane Latimer, Manuela L Ferreira, Paul Lam

**Affiliations:** 1 The George Institute for International Health, The University of Sydney, Sydney, Australia; 2 The George Institute for International Health, The University of Sydney, Sydney, Australia; 3 Faculty of Health Sciences, The University of Sydney, Sydney, Australia; 4 School of Public Health and Community Medicine, The University of New South Wales, Sydney, Australia

## Abstract

**Background:**

Low back pain persisting for longer than 3 months is a common and costly condition for which many current treatments have low-moderate success rates at best. Exercise is among the more successful treatments for this condition, however, the type and dosage of exercise that elicits the best results is not clearly defined. Tai chi is a gentle form of low intensity exercise that uses controlled movements in combination with relaxation techniques and is currently used as a safe form of exercise for people suffering from other chronic pain conditions such as arthritis. To date, there has been no scientific evaluation of tai chi as an intervention for people with back pain. Thus the aim of this study will be to examine the effects of a tai chi exercise program on pain and disability in people with long-term low back pain.

**Methods and design:**

The study will recruit 160 healthy individuals from the community setting to be randomised to either a tai chi intervention group or a wait-list control group. Individuals in the tai chi group will attend 2 tai chi sessions (40 minutes)/week for 8 weeks followed by 1 tai chi session/week for 2 weeks. The wait-list control will continue their usual health care practices and have the opportunity to participate in the tai chi program once they have completed the follow-up assessments. The primary outcome will be bothersomeness of back symptoms measured with a 0–10 numerical rating scale. Secondary outcomes include, self-reports of pain-related disability, health-related quality of life and global perceived effect of treatment. Statistical analysis of primary and secondary outcomes will be based on the intention to treat principle. Linear mixed models will be used to test for the effect of treatment on outcome at 10 weeks follow up. This trial has received ethics approval from The University of Sydney Human Research Ethics Committee. HREC Approval No.10452

**Discussion:**

This study will be the first trial in this area and the information on its effectiveness will allow patients, clinicians and treatment funders to make informed choices regarding this treatment.

**Trial Registration:**

This trial has been registered with Australian New Zealand Clinical Trials Registry. **ACTRN12608000270314**

## Background

The lifetime prevalence of low back pain (at least one episode of LBP in a lifetime) in developed countries is reported to be up to 85% [1]. A significant number of these patients develop chronic/long-term low back pain, defined as pain persisting for more than 3 months [2,3]. Additional to their pain these patients' health problems typically include reduced physical function and psychological distress [4]. In Australia, it has been reported that one in ten Australians suffer from long-term low back pain [5] and it was announced in 2004 as the most prevalent and single most costly musculoskeletal disorder [6]. These data are consistent with that of other industrialized countries worldwide. In the USA, spinal disorders are the most common cause of activity limitation in people under 45 and account for 57.1% of all musculoskeletal impairments in people up to 65 [7]. Similarly in the UK and Sweden, low back pain was estimated to be the largest single cause of work absence [8].

There are a multitude of treatments used in clinical practice to treat low back pain, with little consensus amongst clinicians as to which is the best approach. A recent review of commonly used therapies for chronic low back pain showed that psychological interventions (including cognitive-behavioral therapy and progressive relaxation), interdisciplinary rehabilitation, spinal manipulation, and exercise therapy are all moderately effective compared with placebo or sham therapies [9]. Exercise therapy is relatively inexpensive, easily accessible and widely used in clinical practice to promote an active approach to pain management. However, exercise therapy encompasses a heterogeneous group of interventions [10-12] that vary in type, frequency and duration. [13-16]. While the most effective approach remains uncertain, a list of exercise therapy components associated with best outcome was generated via a meta-regression analysis conducted as part of a Cochrane review in 2005. Several of these components, such as supervision, stretching and strengthening are features of, or can be easily incorporated into, tai chi programs [17].

Tai chi is a low-impact form of exercise of low to moderate intensity that originated in China and consists of slow controlled sequential movements combined with deep diaphragmatic breathing. It has been used as part of Traditional Chinese Medicine for many years and has recently become the focus of scientific research exploring its health benefits. To date, the research has shown programs to be safe [18]and improve physical measures such as pain, self-reported disability, physical performance and falls recurrence [19-21]. There have also been positive effects shown for a variety of psychological indicators such as depression, anxiety, relaxation, concentration, and self-efficacy [22-25]. Although, the bulk of this research is of low methodological quality and reports on small samples, the results thus far are promising and tai chi deserves consideration as an option in the management of other chronic conditions.

We propose to conduct the first randomized-controlled trial of tai chi exercise for long-term low back pain. If the program proves beneficial, this will provide a low-cost and safe alternative for health professionals in the management of this health problem.

## Methods and design

### Participants

Individuals will be recruited via community advertisements. Interested individuals will be screened via telephone by one of the investigators to determine eligibility using the inclusion/exclusion criteria listed in Table 1. All participants require a minimum self-reported score of "moderate" pain or "moderate" activity limitation due to pain as determined by items 7 or 8 on the Short Form-36 item (SF-36) Health Survey (version 2). They must have a diagnosis of "non-specific low back pain +/- leg pain" and be appropriate for exercise management of their back pain measured by the telephone screening questionnaire. If the individual scores positive on any of the telephone screening items a clinical exam and/or medical review will be conducted by one of the investigators who is also a registered physiotherapist to rule out any serious spinal pathology or contraindication to exercise. Individuals suspected of having a serious spinal pathology or any contraindication to exercise will be referred to their medical practitioner for review. Once cleared by their medical practitioner they will be reconsidered for inclusion in the trial. Potential participants will be screened over the phone using three standardized forms: an inclusion/exclusion criteria sheet, a Physical Activity Readiness Questionnaire (PAR-Q) [26] and a pathology screening questionnaire. Participants that meet all the inclusion criteria will have the trial explained to them in detail and then be asked to provide consent before being enrolled into the trial.

**Table 1:** Inclusion and exclusion criteria for the TAI CHI study

*Inclusion criteria*	
Age	18 to 70 years
Diagnosis	Non-specific low back pain +/- leg pain.
Pain duration	At least 3 months duration.
Pain qualifier	A score of "moderate" or higher on item 7 or 8 of the SF-36.
Usual health care	Current health care regimen stable for at least 4 weeks and individual agrees not to seek changes to health care regimen during the 10 week trial.
Language	English speaking and English literate.
Residential status	Expects to continue residing in Sydney for study duration.

*Exclusion criteria*	

Pathology	Suspected or confirmed serious spinal pathology (fracture, metastatic, inflammatory or infective diseases of the spine, cauda equina syndrome/widespread neurological disorder).
	Nerve root compromise (2 of strength, reflex or sensation affected for same nerve root)
Past medical procedures	Spinal surgery
Current medical status	Scheduled for major surgery during treatment
Pregnancy	Suspected or confirmed pregnancy
Contraindications	Any of the contraindications to exercise listed on the PAR-Q
Work status	If participant's back pain is compensable, all relevant treatment parties need to agree to Tai Chi treatment.
Language	Unable to speak, read and write English.
Previous tai chi experience	Participation in a tai chi program within the last 6 months.

*Temporary exclusion criteria*	

Current health care	If participant has started a new treatment/intervention for their back symptoms within 4 weeks prior to eligibility assessment, the individual is temporarily excluded from participating in the study. The individual will be advised that once the new course of treatment has either finished or has become part of their regular health care routine for more than 4 weeks, and they are still experiencing low back pain symptoms they can contact the project leaders again to participate in the trial.

#### Data collection location

All volunteers will be recruited from the Sydney region, all outcome assessment and tai chi sessions will be conducted at participating community venues within the Sydney metropolitan area.

### Interventions

#### Tai chi program rationale

Currently, the mechanisms underlying tai chi have not been well established. However, tai chi increases flexibility and improves lower body strength, two components that have been associated with successful exercise programs for long-term low back pain [27]. Furthermore, previous research has consistently shown tai chi to have positive effects on pain, physical function and quality of life in other populations with chronic conditions [16-18]. The 4 main styles of tai chi; Chen, Wu, Yang and Sun all contain the same basic principles of slow, continuous movements combined with deep diaphragmatic breathing and maintenance of an upright posture. Compared to the other styles, Sun is characterized by less knee flexion which results in decreased stepping distance and more follow-up steps. These characteristics were found to place a lesser burden on the knee and ankle joints [20]. The Sun style form we have chosen for this study is one that is currently practised and promoted by arthritis foundations worldwide and is also being recommended in clinical practice. The form has been designed by Dr Paul Lam, general practitioner and tai chi master and includes 21 moves and can be viewed on DVD.

#### Tai chi instruction method

Tai chi sessions are typically held in group format. Instruction is both verbal and visual. A systematic approach to teaching tai chi has been developed by Dr Paul Lam [28]; it is known as the *Stepwise Progressive Teaching Method*. This approach is used world-wide by certified instructors in the teaching of Tai Chi for Health Forms; *Tai Chi for Diabetes, Tai Chi for Arthritis, Tai Chi for Back Pain *and *Tai Chi for Beginners 24 forms*. This method promotes breaking down the tai chi form into the individual movements and teaching them separately, starting with the first move and moving on to the next in a sequential manner. This method does not change the purity of the tai chi form in any way but provides a standardized teaching method. Thus, our study will use the *Stepwise Progressive Teaching Method *to ensure repeatability. A detailed description of the approach is attached in the Appendix.

#### Tai chi dosage and class format

Participants in the intervention group will receive 18 × 40 min sessions over a 10 week period; 2 sessions/week for the first 8 weeks, 1 session/week for the remaining 2 weeks. The treatment sessions are designed to become less frequent over time to represent what is available in the community and encourage independence and continuation of tai chi exercise when therapy is complete.

Each tai chi session has a lesson plan for the instructor to follow which is located in the instructor's manual. The lesson plan includes a checklist of the topics each session will focus on. The instructor will use the lesson plan to conduct the tai chi class and check off that each topic has been covered. Each lesson plan is sufficiently descriptive to allow an independent tai chi instructor to replicate the tai chi class. An exercise log will be provided for each participant to record their home tai chi practice. The home log is designed as a motivational tool for each participant to record their home practice goals and achievements for each week. At the beginning of each week, the participant will fill out how much time they intend to practise each day. They then take their diary home and record their actual practice time per day. The home logs will then be used in the class sessions to discuss any barriers that may have arisen in achieving their goals. Each tai chi group has a session attendance sheet that the instructor completes at the beginning of each class.

#### Other interventions

A bi-weekly questionnaire will be sent via email to participants in both treatment and control groups. The questionnaire will collect the health services accessed by the participant in the prior 2 weeks. Examples of health services include physiotherapy, chiropractic, acupuncture, massage and GP visits related to back symptoms. If no response is received within 24 hrs, a second email will be sent as a follow-up. If no response is received within 48 hrs, the participant will be contacted via phone. Participants in both treatment and control groups will be asked not to seek any new treatments for their chronic low back pain and where possible not to change current treatment during the trial intervention period.

#### Safety

If a participant is concerned about his or her condition during the study, they will be referred to discuss the situation with one of the investigators who is a registered physiotherapist. Where appropriate, the physiotherapist will refer the patient to a medical practitioner.

### Objectives

#### Study aim

The primary aim of this project is to establish whether tai chi exercise is more effective than usual care in the management of long-term low back pain. Individuals who have low back pain for at least 3 months will be randomly assigned to either a tai chi exercise group or to a wait-list control group. The tai chi group will participate in a 10 week sun-style tai chi program consisting of 18 × 40-min group sessions led by a certified instructor. The primary study outcome will be change in an individual's self-reported level of "bothersomeness of back symptoms" as measured by an 11pt numerical rating scale. Secondary outcomes will include change in self-reported intensity of pain, physical function, global perceived effect of treatment and health related quality of life measures.

#### Primary Hypotheses

The effect of a 10 week tai chi exercise program and usual health care is greater than that of usual health care alone where effect is measured in terms of bothersomeness of back symptoms, disability and global perceived effect of treatment and quality of life.

### Outcomes

The primary endpoint with respect to the effectiveness of a 10 week tai chi program for long-term low back pain is bothersomeness of back symptoms measured on an 11 point numerical rating scale. This scale is being more widely used in back pain research and has been shown to be sensitive to change [29-31]. Secondary outcomes are self-reported disability, health-related quality of life and global perceived effect of treatment. The treatment efficacy variables will be measured at baseline and 10 weeks.

#### Specific outcome measures

• *Bothersomeness of back symptoms *measured by the Bothersomeness (0–10) Numerical Rating Scale [32].

• *Generic pain-related disability *measured with the Pain Disability Index [33].

• *Back-specific pain-related disability *measured with the Quebec Back Pain Disability Scale [34].

• *Patient-specific pain related disability *measured with the Patient Specific Functional Scale [35,36].

• *Health-related Quality of Life *measured with the SF-36v2 Health Survey [37].

• *Coping Strategies *measured with the Coping Strategies Questionnaire [38].

• *Global perceived effect of treatment *measured with the Global Perceived Effect Numerical Rating Scale (-5 to +5) [35,36].

#### Additional Measures for Supplementary Clinimetric Analyses

• Roland Morris Disability Questionnaire 24 item [39].

• Patient Specific Functional Scale version 2

#### Demographic Measures

• A demographic data sheet including; age, gender, height, weight; ethnicity, religion, marital status, smoking status, previous episodes of back pain, previous treatments for back pain, current treatments and current exercise activities will be completed at baseline assessment.

#### Usual Care Measures

• A bi-weekly list of the health services accessed by the participants in both control and intervention group will be collected via email.

#### Process Measures

• In-class attendance sheets (adherence to treatment)

• Class lesson-plan checklist (standardized instruction)

#### Data integrity

The integrity of trial data will be monitored by regularly scrutinizing data sheets for omissions and errors.

### Sample Size

We have designed the study to detect a clinically important difference of 1.5 units on the 0–10 bothersomeness scale (estimate for SD = 2.35), 1 unit on the 0–10 pain numerical rating scale (estimate for SD = 2), 1 unit on the 0–10 patient specific functional scale (estimate for SD = 1.8); 1 unit on the 0–10 Global Perceived Effect Scale (estimate for SD = 1.65), 4 units on the 24 item Roland Morris Disability Questionnaire (estimate for SD = 4.9). We have taken the SD estimates from trials that recruited a similar patient cohort [3,29,40]. Given these considerations we have chosen to design the study to detect an effect size of 0.5 SD. Based upon the results of a similar trial by our group [3] we have allowed for 15% non-compliance with treatment, 15% loss to follow-up, and assumed a correlation between baseline and change scores of outcomes of 0.5. Taking these factors into account with specifications of alpha = 0.05, power = 0.80 a sample size of 77 participants per group is required to detect an effect size of 0.50 SD [41]. Accordingly we will recruit 80 participants per group or 160 participants in total to allow for treatment in blocks of 8. Blocks of 8 allow us to randomize 4 subjects at the one time to the Tai Chi group and so have a sufficiently large class.

As a secondary analysis we will evaluate predictors of response to treatment. We estimate that it would require ~640 subjects to detect an interaction effect of the same magnitude as the main effect. We do not have the resources for such a study. Accordingly we acknowledge that we may miss an interaction effect in our analyses. Brookes and colleague's simulations demonstrate that when testing for effect modification, trials have the same power to detect an interaction effect that is twice the size of the main effect [42].

### Randomization

#### Sequence generation and allocation concealment and implementation

The allocation sequence will be generated using the random number function in Excel. The allocation sequence will be generated by an investigator not involved in assessment and treatment codes placed sequentially into sealed opaque envelopes. Baseline assessment will be conducted prior to group allocation by an investigator blind to the allocation sequence. Following assessment, participants will be issued a sealed envelope containing their group allocation and those in tai chi group scheduled to attend their first class within one week.

The tai chi intervention includes a series of movements to be taught sequentially and is taught in a group format. For reasons of practicality, the randomization code was developed using a computer random number generator in a block size of 8. This allows us to allocate 4 subjects to the tai chi class, and 4 to control for each block. This assists scheduling of tai chi classes which are held in groups.

#### Blinding

Participants will not be blinded to treatment as the control group is not receiving any intervention in addition to their usual care. The investigator responsible for delivery of treatment will be blinded to allocation sequence and will become unblinded as participants enter treatment group. The investigators responsible for the monitoring of treatment and attending to clinical issues that arise during the conduct of the study will be unblinded. Assessment is self-report questionnaire completed by participants which precludes blinding of assessment. The investigators responsible for data analysis will be blinded to treatment allocation for the duration of trial.

### Timeline

Timeline and flow from screening through randomization and follow-up assessments are shown in Table 2. Participants who successfully meet the inclusion criteria will be asked to attend an initial group session to complete the baseline assessment including a series of questionnaires. After all outcome measures are completed and checked by the blinded assessor for any missing or incomplete data, the participants will be randomized into the intervention or control group. The blinded assessor will score and enter the outcome measure data into an SPSS spreadsheet. The tai chi instructor will organize the intervention group to begin their first tai chi session within one week of randomization. The participants in the tai chi intervention group will continue to attend tai chi sessions 2 times per week for 8 weeks, followed by 1 time per week for 2 weeks. The control group will be asked to continue with their usual health care for the following 10 weeks and given the opportunity to participate in a tai chi program identical to the one in the intervention group, upon completion of the follow-up assessment. During the 10 week intervention both groups will receive a bi-weekly questionnaire via email. The questionnaire collects data on the health care services accessed by the participant in the prior 2 weeks. Upon completion of the 10 week intervention, all participants will attend the follow-up assessment in a group format. To maximize attendance at the follow-up assessment each participant will be contacted by phone, email or a sms text to confirm attendance 1 week prior to follow-up assessment. An additional reminder will be made 1 day prior to follow-up assessment. The outcome data will be scored and entered into an SPSS spreadsheet within 3 days by one of the blinded investigators.

**Table 2:** Participant flow and timetable from screening to follow-up assessment

**Timeline**	**Action**	
Week 0	Telephone screen conducted by one of the investigators	receive patient information statement verbal consent complete telephone screening questionnaire to determine eligibility eligible individuals are invited to attend baseline assessment conducted in groups of 8 individuals
Week 1	Baseline Assessment administered by blinded assessor	sign patient informed consent complete demographic data complete baseline questionnaires
	Randomization	*generation*: using sealed opaque envelopes containing allocation sequence generated by a computer random number function *allocation*: the unblinded tai chi instructor will assign participants to one of two groups the tai chi intervention group or the wait-list control group
Week 2 – 11	Intervention conducted by un-blinded investigator	*Tai chi group*: 2 × 40 min group sessions/week for 8 weeks 1 × 40 min group session/week for 2 weeks
		*Wait-list control group*: Continue with pre-established usual health care After follow-up assessment is complete, participant can join in tai chi program identical to that of intervention
	Assessments during intervention administered via email by blinded assessor	Both groups will receive a bi-weekly questionnaire asking them to list the health care services they have used for their back symptoms
Week 12	Follow-up Assessment administered by blinded assessor	administered by blinded assessor complete follow-up questionnaires after assessment, blinded assessor will leave and tai chi instructor will hand-out certificates of completion to tai chi group and schedule wait-list control group for tai chi program

### Statistical Methods

#### Primary analysis: treatment efficacy

Statistical analysis of primary and secondary outcomes will be based on the intention to treat principle. The effect of intervention on bothersomeness of pain, pain intensity, function, disability and global perceived effect will be determined using linear mixed models (random intercepts and fixed coefficients) which incorporate terms for treatment, time and the treatment by time interactions. The coefficients of the treatment by time interactions provided estimates of the effects of the tai chi intervention. A treatment effect size will be calculated for each of the follow-up time points and, if there is a statistically significant treatment effect at any time point, we will also calculate number needed to treat (NNT) to achieve pain recovery (pain < 1 out of 10: [43] and 95% confidence intervals.

#### Secondary analyses: Predictors of Response to Treatment

As stated previously, long-term low back pain affects 10% of Australians ranging across a heterogeneous group. It is worthwhile to determine predictive factors of patients in order to determine if tai chi would be a beneficial treatment for that individual.

First, we will assess whether any single baseline predictor can identify patients who respond best to Tai Chi. To limit the chance of Type 1 error a limited list of potential predictors has been determined a-priori on the basis of biological rationale, they are: (bothersomeness of back symptoms score measured with the bothersomeness scale, physical function measured with the *patient specific functional scale*, stress level measured with the DASS-21, anxiety level measured with the DASS-21, and the patients self-reported expectation of treatment. A model will be created for the primary outcome measure, bothersomeness of back symptoms and will include the interaction term between treatment allocation (Tai Chi or control) and baseline predictor, as well as, treatment allocation and baseline predictor as terms on their own. The statistical significance and size of the interaction coefficient will be assessed to determine whether the baseline characteristic is able to identify patients who respond best to the treatment.

Second, we will assess if a combination of baseline characteristics is more effective than any single characteristic at identifying patients who respond best to Tai Chi. A multivariate backwards stepwise linear regression model will be used to assess this question. To be considered as a candidate variable for the multivariate analysis the interaction term in the first model (primary analysis) will be required to be associated with outcome (p < 0.25). We will inspect the correlations between the candidate variables and where any correlation is greater than 0.4 we will select one of the variables based on ease of assessment and psychometric properties. Significant variables will be entered into the multivariate model. A p-value for interaction terms > 0.05 will be used to remove both the predictor and its interaction from the model. We will assess whether the final model can be simplified to create a rule which is appropriate for use in a clinical setting.

## Discussion

This study will evaluate the effects of a 10-week tai chi exercise program on people with long-term low back pain. The study is adequately powered to detect change in the primary and secondary outcomes that include bothersomeness of back symptoms, self-reported disability and physical function and global perceived effect of treatment. The focus of this study is to determine the efficacy of tai chi as an intervention for long-term low back pain; it aims to provide both health care providers and patients with evidence-based advice for treatment planning. To our knowledge this is the largest study specifically designed to examine the effect of tai chi on low back symptoms in people with long-term low back pain.

## Competing interests

We acknowledge a potential competing interest with author Paul Lam who in addition to being a medical practitioner also owns a business, entitled Tai Chi Productions that produces and sells educational material regarding the health benefits of tai chi.

## Authors' contributions

AH, CM, JL and MF equally contributed to all aspects of the methodological design of the study. PL provided expertise regarding dosage and implementation of intervention. All authors read and approved the final manuscript.

## Pre-publication history

The pre-publication history for this paper can be accessed here:


